# Cross-Whisker Adaptation of Neurons in Layer 2/3 of the Rat Barrel Cortex

**DOI:** 10.3389/fnsys.2021.646563

**Published:** 2021-04-28

**Authors:** Yonatan Katz, Ilan Lampl

**Affiliations:** Department of Neurobiology, The Weizmann Institute of Science, Rehovot, Israel

**Keywords:** barrel cortex, whisker stimulation, layer 2/3 cortex, receptive fields, integration, intracellular, *in vivo*

## Abstract

Neurons in the barrel cortex respond preferentially to stimulation of one principal whisker and weakly to several adjacent whiskers. Such integration exists already in layer 4, the pivotal recipient layer of thalamic inputs. Previous studies show that cortical neurons gradually adapt to repeated whisker stimulations and that layer 4 neurons exhibit whisker specific adaptation and no apparent interactions with other whiskers. This study aimed to study the specificity of adaptation of layer 2/3 cortical cells. Towards this aim, we compared the synaptic response of neurons to either repetitive stimulation of one of two responsive whiskers or when repetitive stimulation of the two whiskers was interleaved. We found that in most layer 2/3 cells adaptation is whisker-specific. These findings indicate that despite the multi-whisker receptive fields in the cortex, the adaptation process for each whisker-pathway is mostly independent of other whiskers. A mechanism allowing high responsiveness in complex environments.

## Introduction

The organization of receptive fields in layer 2/3 of the barrel cortex has been studied in rodents mainly using passive stimulation of individual whiskers. In the barrel cortex neurons typically respond primarily to stimulation of a single whisker and somewhat less to neighboring whiskers (Simons, 1985; Simons and Carvell, 1989; Armstrong-James et al., 1992; Bruno and Simons, 2002). Layer 4 cells typically have wider receptive fields than layer 2/3 cells (Bureau et al., 2006; Viaene et al., 2011). Vertically directed axons project excitation from layer 4 cells into layer 2/3 cells of the same column (Lübke et al., 2000). Hence the response in layer 2/3 cells to whisker stimulation is delayed by 2–3 ms relative to the response in layer 4 cells (Armstrong-James et al., 1992). This additional delay supports the notion that layer 2/3 are innervated mostly by cortical cells. However, their response to multiple whiskers may reflect inputs from layer 4 or neighboring columns (Simons, 1985).

Assuming that the synthesis of the wider receptive fields of layer 2/3 emerges in the cortex, the adjacent whisker response of these cells can reflect inputs from the same column via multi-whisker layer 4 cells and/or from adjacent barrels through horizontal connections. Some proposed that the whisker-trigeminal system is a labeled-line pathway, where subcortical structures relay to layer 4 cortex only principal whisker (PW) inputs and adjacent whisker (AW) responses are therefore synthesized by intracortical interactions (Armstrong-James and Callahan, 1991; Armstrong-James et al., 1991; Fox et al., 2003). Others found that AW responses in layer 4 are independent of intracortical interactions, reflecting direct thalamic inputs that relay both PW and AW inputs (Simons and Carvell, 1989; Goldreich et al., 1999; Urbain and Deschênes, 2007). For layer 2/3 cells, most studies agreed that suprathreshold excitation spreads horizontally within layer 2/3 into the adjacent cortical columns and subsequently across the entire barrel field. *In vivo* whole-cell recordings suggest that most layer 2/3 pyramidal cells respond with large EPSPs upon deflection of a single whisker and have subthreshold receptive fields broader than those of layer 4 spiny neurons (Brecht and Sakmann, 2002; Brecht et al., 2003), suggesting intracortical integration. Based on this hypothesis, the adjacent whisker response is independent of layer 4 activity of the principal column. In other words, if adjacent whisker response in layer 2/3 emerges from a neighboring barrel, applying repetitive stimulation to the PW, which leads to adaptation, should not affect the cell’s response to stimulation of an adjacent whisker. On the other hand, if the multi-whisker receptive field is inherited from layer 4 cells, a cross-adaptation effect should be observed, leading to attenuation of the response to AW stimulation following adaptive stimulation of the PW. Considering the latter possibility, cross-whisker adaptation might not be always observed: when a test AW stimulation is applied after several stimuli were applied to the PW, due to their low firing response, the synapses connecting layer 4 to layer 2/3 may recover from the adaptation process, leading to a large response to the test stimulation. To overcome this issue we examine the adaptive interactions using an interleaved stimulation of the PW and AW. We assume that such interleaved stimulation leads to a high level of activity in layer 4, maintaining synapses common to the two stimulated whiskers at a depressed state. Such stimulation therefore allowed us to track the interactions over time as adaptation progresses. We hypothesized that the convergence of different whiskers’ pathways at the level of layer 4 should result in a stronger interaction during adaptation compared to the expected interactions when a synthesis of the receptive fields occurs at the level of layer 2/3.

We intracellularly recorded the response of layer 2/3 neurons to interleaved whisker stimulation of the PW and AW and evaluated the degree of interactions between them due to adaptation. We found no interactions for most layer 2/3 cells. Significant interactions were found only in about one-quarter of the tested whiskers, where profound adaptation induced by repetitive stimulation of one whisker caused a significant reduction in the response of a second whisker. No physiological parameters such as latency or response amplitude were related to the level of interactions.

## Materials and Methods

### Ethics Statement

All surgical and experimental procedures were performed in accordance with the regulations of The Weizmann Institute Animal Care and Use Committee (IACUC App.Num. 00470109-1).

### General

Adult female Sprague–Dawley rats (*n* = 18) were anesthetized with halothane (0.5–1.0%), tracheotomized, and ventilated. Heart rate, temperature, and expired CO_2_ were monitored continuously. Intracellular recordings were made from barrel cortex layer 2/3 neurons using glass micropipettes (5–9 MΩ) filled with an intracellular solution containing in mM: K-gluconate, 136; KCl, 10; NaCl, 5; HEPES, 10; MgATP, 1; NaGTP, 0.3; phosphocreatine, 10; mOsm, 310. For each recording, the principal whisker (PW) and the adjacent whisker (AW) having the highest amplitude were mechanically deflected in the PW preferred direction using a piezoelectric stimulator (for details see Katz et al., 2006).

In these experiments, intracellular whole-cell patch recordings were used and membrane potential was not compensated for the junction potential (∼12 mV). Signals were amplified using Axoclamp-2B (Molecular Devices, Palo Alto, CA, United States) and low passed at 4 kHz before being digitized at 10 kHz.

### Stimulation Protocol

**FIGURE 1:**
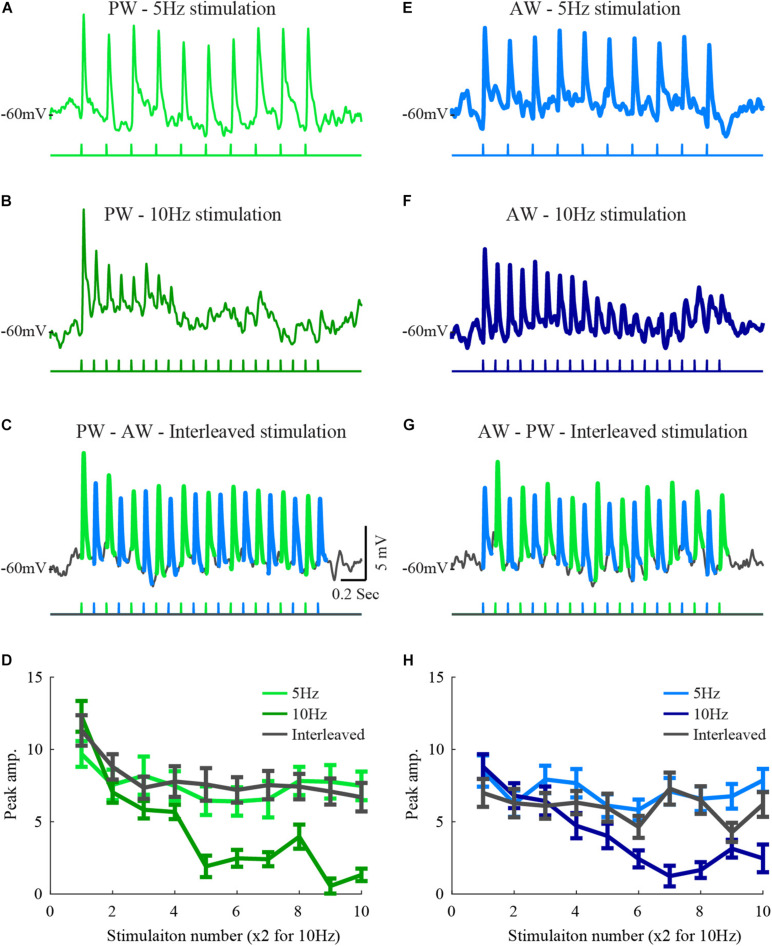
Responses of a layer 2/3 cell to stimulation of two whiskers during interleaved stimulation. **(A)** The average response of a layer 2/3 cell to 5 Hz stimulation of the principal whisker (stimulation pattern is depicted below the trace). **(B)** The average response of the same cell to 10 Hz stimulation of the PW principal whisker. **(C)** The average response to stimulation of the principal whisker and to the adjacent whisker under interleaved stimulation (the trace was segmented by colors to depict the response to the two stimulated whiskers. **(D)** The average peak amplitudes of the three stimulation patterns in **(A–C)** are depicted in light-blue for the 5 Hz stimulation, blue for the 10 Hz, and in dark gray for the response to the principal whisker during the interleaved stimulation. **(D–H)** Same conventions as in **(A–D)** for responses to the adjacent whisker. The scales are similar for all plots.

For control stimulation, a 5/10 Hz train of 10/20 stimuli was used for each of the two whiskers. To estimate the interaction between whiskers, 10 Hz interleaved-stimulation was presented where each whisker was actually stimulated at 5 Hz (see Figure 1). These responses were compared to either 5 Hz or 10 Hz stimulation trains delivered to each whisker alone. During each trial, a 3 s inter-train interval was used.

### Response Quantification and Adaptation Index

We have quantified a number of response properties such as the amplitude, latency, rise-time, and adaptation index. The response amplitude was defined as the maximal amplitude relative to baseline within a 50 ms window following the stimulation. The latency to response was defined as the time from stimulus onset to 10% deviation of the averaged membrane potential from baseline (PSP onset) was defined as the latency to the response. The response rise-time was quantified as the time measured from 10% to 90% of the response amplitude.

The adaptation index (AI) was quantified according to the following equation:

$$A\,I=1-\frac{mean\left(R_{\left(n-1\right)}:R_{n}\right)}{R_{1}}$$

Explicitly, this is one minus the ratio between the mean of the last two responses [R_(n–1)_ and R_(n)_] and the first responses (R_1_) was calculated. The number of stimulations during 5 and 10 Hz was 10 and 20, respectively.

### Calculation of the Interactions Between Whiskers and Adapted-State Response

Here we define the cross-whisker adaptation under interleaved stimulation. When a response of a cell to the deflection of one whisker is affected by the preceding whisker deflection of another whisker, it is defined as an interaction. The interaction can either be facilitatory or inhibitory. To measure the interaction for a given stimulation (i), the given formula was used:

$$I\,n\,t\,e\,r\,a\,c\,t\,i\,o\,n\,\left(i\right)=1-\frac{R_{i\,n\,t\,e\,r\,l\,e\,a\,v\,e\,d}\,\left(i\right)-R_{10\,H\,z}\,\left(i*2\right)}{R_{5\,H\,z}\,\left(i\right)-R_{10\,H\,z}\,\left(i*2\right)}$$

R = Averaged EPSP

where R_*Interleaved*_(i) denotes the ith response of the cell to the stimulated whisker during the interleaved stimulation of the relevant whisker. R_10 *Hz*_(i^∗^2) denotes the response of the cell during 10 Hz whisker stimulation; because the stimulation frequency is double that during 5 Hz stimulation, the response to the (i^∗^2) stimulation is measured. R_5 *Hz*_ denotes the control response during 5 Hz stimulation. The change in response from 5 Hz to 10 Hz provides a reference for the interactions.

An index of 1 will be obtained for full interaction (the amount of adaptation for the interleaved stimulation is identical to that expected from a single whisker stimulation) and 0 if the response was not affected by interleaving it with stimulating the other whisker. If the response to 10 Hz stimulation is weaker than the response to 5 Hz stimulation and the interleaved response has an intermediate value in between, then the interaction value can vary between 0 when there is no interaction to 1 for a strong interaction. Thus, response to whisker stimulation which was significantly suppressed during interleaved stimulation was termed “interacting.” According to the level of interactions of cells for responses to stimulation of the two whiskers, cells were divided into three groups: 1. Interacting, 2. Non-interacting, 3. Unidirectional-interacting when only one whisker affected the response of the other one and not vice versa.

The adapted-state response was defined as the mean response of the last two deflections in 5 Hz or 10 Hz train stimuli.

## Results

### Recorded Population

Intracellular whole-cell patch recordings were performed in layer 2/3 of the barrel cortex of anesthetized rats. Overall, 28 cells were recorded in 18 animals. For each cell, the principal whisker (PW) and most responsive adjacent whisker (AW) were stimulated. The average recording depth was 411 ± 117 μm and the mean latency whisker responses were 7.7 ± 0.3 and 8.7 ± 0.3 ms (for PW and AW), corresponding to layers 2/3 in the rat (Katz et al., 2006).

### The Response of Layer 2/3 Cells to 5 Hz, 10 Hz, and Interleaved Stimulation of the Principal and Adjacent Whiskers

Feed-forward inputs to layer 2/3 ascend mainly from layer 4 (Fanselow et al., 2001; Helmstaedter et al., 2009). If layer 4 cells integrate inputs from different whiskers and if adaptation results from local mechanisms such as short-term synaptic plasticity, strong interactions are expected in response to the interleaved stimulation. However, our former results from layer 2/3 recordings showed only weak, but significant, interactions between the inputs from two different whiskers (Katz et al., 2006). In this former study, test stimulation to a whisker was delivered following repetitive stimulation of its neighboring whisker. We reasoned that due to the low firing rate of layer 4 cells, in particular toward the end of the stimulation train, the ascending synapses to layer 2/3, common to the two whiskers, will recover from depression and thus restore the ability to respond upon subsequent stimulation of the neighboring whisker. Here we used an interleaved stimulation protocol to keep the ascending pathway at a depressed state and thus allow evaluation of the interactions while stimulating the two whiskers.Hence, this form of stimulation allows testing the interactions between whiskers pathways during the adaptation process, well before the response is fully adapted. For each cell, we tested the adaptation to PW or AW stimulation at two frequencies. An example cell is shown in Figure 1. Neuronal responses of this cell to 5 Hz stimulation were weakly adapted (AI = 0.22 ± 0.03, Figures 1A,E). Adaptation was more pronounced at 10 Hz stimulation (*p* < 0.005, AI = 0.47 ± 0.02, Figures 1B,F). To test for interactions, we used interleaved stimulation. Each of the two whiskers was stimulated at 5 Hz with half a cycle shift between the trains resulting in effective 10 Hz stimulation input at the recorded cell (Figures 1C,G).

Interleaved stimulation in this example only weakly affected the adaptation pattern that is expected from single whisker stimulation at 5 Hz. This was observed by comparing the responses to stimulation of each whisker in the interleaved average response to those evoked by the 5 Hz stimulation (compare traces in Figures 1A,E,C,G, the pattern of stimulation is depicted below the averaged membrane potential traces). The amplitude of the responses during interleaved stimulation closely matched the response to 5 Hz stimulation, indicating that the presence of stimulation of the neighboring whisker did not affect the time course and magnitude of the adaptation when considering stimulation of each whisker alone. The average peak response amplitudes during each stimulation protocol were measured (Figures 1D,H), and used for population analysis. Since the number of whisker deflections during 10 Hz stimulation was double that during 5 Hz, only response amplitudes for odd deflections are presented.

**FIGURE 2:**
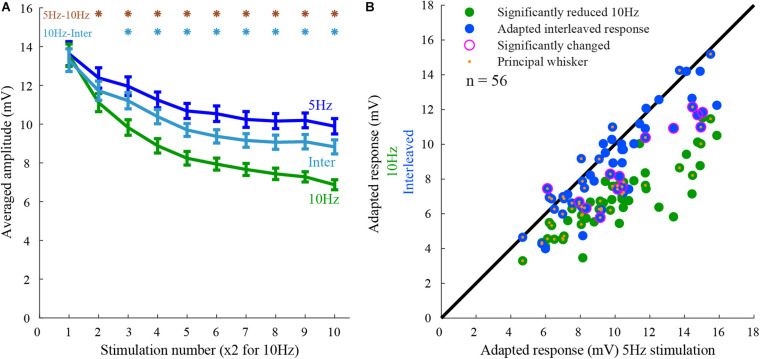
Population response to 5 Hz, 10 Hz, and interleaved stimulation. **(A)** The peak amplitude of the responses to the three stimulation conditions (5Hz, 10Hz, and interleaved). Note that cells exhibited adaptation from the second stimulus onward. The degree of adaptation during the interleaved stimulation was closer to that for 5 Hz stimulation than for the 10 Hz. The responses to 10 Hz stimulation were significantly different from the others, as marked by the asterisks (rank-sum, *p* < 0.05). **(B)** Responses of layer 2/3 cells (each point represents a single whisker test) to whisker deflection at 10 Hz and interleaved stimulation protocols are compared to that obtained by 5 Hz stimulation. The adapted-state response to stimulation of each whisker obtained by the control stimulation pattern (5 Hz) was on average larger than the response to 10 Hz stimulation (green) and interleaved stimulation (blue). Large magenta circles indicate a significant difference from the response to 5 Hz stimulation. Orange dots mark the principal whiskers.

The population peak response amplitudes to 5 Hz (Figure 2A blue line), 10 Hz (Figure 2A, green line), and the interleaved stimuli (Figure 2A, cyan line) were averaged across the population (*n* = 56 whiskers, 28 cells). The responses to 10 Hz stimulation were reduced by 50% and were significantly smaller than the responses to 5 Hz (27% reduction) or interleaved stimulations (36% reduction), (rank-sum *p* < 0.05). Hence, on average the responses to the two whiskers interacted, but the effect was not significant and much smaller than from the expected full interaction.

If inputs evoked by stimulation of one whisker undergo adaptation independently of those evoked by the other whisker, stimulation of a second whisker will not affect its response as previously found in layer 4 cells (Katz et al., 2006). In that case, one would expect that the responses during the interleaved stimulation will be equal to the responses during 5 Hz stimulation, having no interactions. On the other hand, if inputs from both whiskers ascend along the same pathway and undergo adaptation after the two pathways converged, the interleaved response will be similar to the response during 10 Hz stimulation, exhibiting strong interactions. We evaluated the interactions during adapted-state responses (see section “Materials and Methods”). Surprisingly, on average, cross-whisker interactions during adapted-state responses were not significantly different from the average control response to 5 Hz stimulation (Figure 2A). The interleaved responses were significantly different in only 14 out of 56 stimulated whiskers (rank-sum *p* < 0.05, 28 cells). All but one of these responses were significantly smaller than the control responses (Figure 2B, outer purple circles).

**FIGURE 3:**
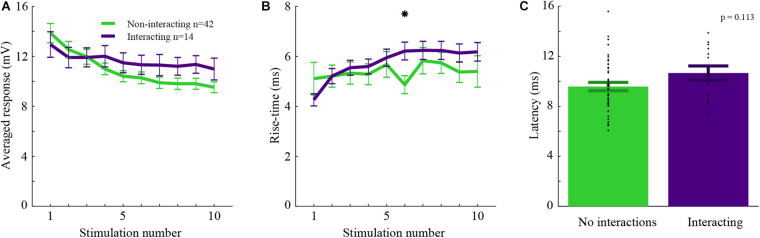
Interacting and non-interacting whisker-inputs have similar response parameters. **(A)** The averaged response amplitudes during 5 Hz stimulation of interacting (purple) and non-interacting whisker-inputs (green). **(B)** The averaged rise time of the interacting and non-interacting whisker-inputs was increased as stimulation progressed, it was significantly different only during the 6th stimulation. **(C)** The average latencies of interacting and non-interacting whisker-inputs were not significantly different. Error bars represent SEM in all panels.

For whisker-inputs exhibiting significant interacting responses (*n* = 14), synaptic inputs might have different properties compared to those that exhibit no interactions (*n* = 42). For example, two interacting whisker-inputs might have similar synaptic response properties, suggesting that they share the same pathway and thus undergo adaptation after the point of convergence. Alternatively, the affected input might evoke a smaller response due to the recruitment of a subpopulation of afferents evoked by stimulation of the other input. In this case, such a difference will lead to an asymmetrical cross-whisker adaptation effect. Thus, various properties such as the amplitude, rise-time, and latency of interacting versus non-interacting inputs were measured (Figure 3). The response amplitudes of interacting whisker-inputs were not significantly different from that of non-interacting whisker-inputs (rank-sum, *p* > 0.05), even though during train stimulation only the responses of non-interacting whisker-inputs were significantly reduced (Figure 3A, rank-sum, *p* < 0.005).

The rise time of both groups was not significantly different (except for the sixth stimulus). Still, during repeated stimulation, non-interacting inputs had a significant increase in the rise time (Figure 3B). Another essential property of the responses is latency. If the input of either group arrives via different pathways, then their latency might be different. However, the average latency for both groups’ responses was similar (10.7 ± 0.8 and 9.6 ± 0.3 ms, respectively, rank-sum, *p* > 0.05).

**FIGURE 4:**
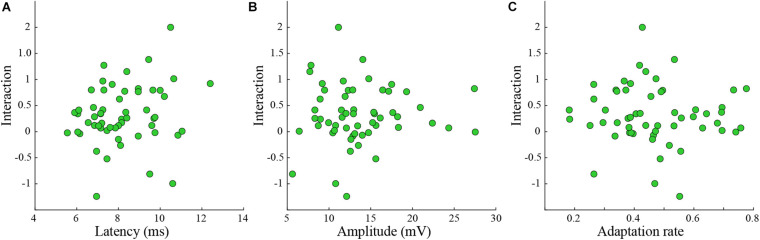
The interaction level was not correlated to various response parameters. **(A–C)** Interaction was not correlated either to latency **(A)**, amplitude **(B),** or adaptation index **(C)** (rank-sum *p* > 0.05 for *n* = 56 tested whiskers).

The division into groups causes information loss (Felsenstein and Pötzelberger, 1998; Taylor and Yu, 2002; Altman and Royston, 2006; Bennette and Vickers, 2012). Hence the statistical power to detect a relationship between the variable (i.e., latency, amplitude, and adaptation rate) and the interaction is reduced. Thus, we tested if the interaction level was related to the latency, the amplitude, or the adaptation rate of the response (Figure 4). Again, we found that the interaction was not correlated to any of these parameters (Figures 4A–C).

**FIGURE 5:**
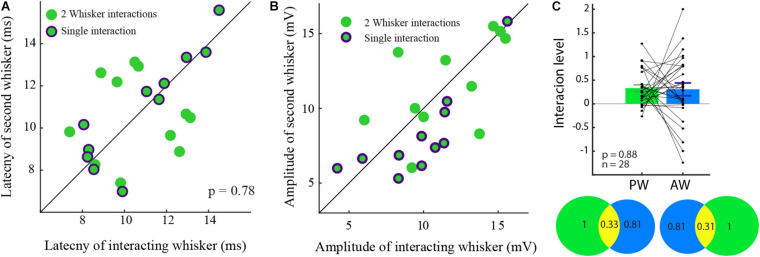
Similar latency and amplitude for unidirectional and interacting cells. **(A)** The latency to the response of the two cell groups was not significantly different. **(B)** The amplitude ratio of the two cell groups was similar. **(C)** The level of input-interaction between the whiskers was not dependent on the response amplitude. The level of interactions between PW and the AW evoked inputs in the bar plot is summarized in the Venn diagram showing the interactions (yellow) between for the PW (green), and the AW (blue) inputs which are reciprocal.

In an attempt to understand the interactions on a single cell level, cells with interactions were divided into two groups by the type of the input-interactions. Cells in which the responses were reciprocally affected (Figure 5, green dots) and cells in which interactions were unidirectional, namely where only one whisker-input was affected (green encircled by purple). Since amplitude and latency might be related to the level of interactions, the ratios of these properties were calculated within the groups. Neither the amplitudes nor the latencies were different between the groups (rank-sum *p* > 0.05).

Specifically, in unidirectional cells (Figures 5A,B green dots encircled by purple), the response amplitude and latency of the non-interacting responses (abscissa) were similar to those of their paired responses (rank-sum, *p* > 0.05, in both properties).

To evaluate whether the level of interactions depends on the amplitude of the response, the level of input interaction between each pair was reciprocally quantified. The level of interaction was found not to be dependent on the response amplitude (0.32 ± 0.4, *p* > 0.5).

To further check if the level of the interaction is related to the identity of the stimulated whisker (PW/AW), we compared the level of interaction between the inputs evoked by stimulation of the PW and the AW. We found that the interactions of the PWs and the AWs inputs were similar and reciprocal (Figure 5C; rank-sum *p* > 0.5). On the Venn diagram, one can see that stimulation of the PW reduced the AW whisker responses by 33% on average (Figure 5C, bottom left circles) and stimulation of the AW reduced the response to stimulation of the PW by 31% (right) on average.

## Discussion

We evaluated the synaptic interactions between inputs arriving from the principal and the adjacent whisker in layer 2/3 cortical cells of anesthetized rats. Interleaved stimulation interactions were assessed according to the expected interactions from the adaptation of the responses to 5 and 10 Hz stimulation of each whisker alone. If the response to interleaved stimulation resembles the one that is expected from 5 Hz, no interactions are observed, whereas if it is similar to that obtained under 10 Hz, the two adaptation processes strongly interact. We found that in ∼75% of the tested whiskers, evoked synaptic inputs were not affected by the stimulation of a neighboring whisker, indicating whisker-specific adaptation.

Inputs that were affected had no specific property (such as latency to response, amplitude, or adaptation level) that can predict the existence of the interactions (although some tendency for a longer response latency was found for the interacting, affected whiskers as shown in Figure 3C). The results suggest that the adaptation to whisker stimulation in layer 2/3 cortical cells is primarily independent, further strengthening our previous study (Katz et al., 2006). Yet, the functional role of whisker-specific adaptation in the detection and discriminability of sensory stimuli is unclear. We suggest that during natural whisking behavior, this specificity enhances both aspects of perception by routing sensory signals independently to a different population of neurons, without being affected by the recent history of stimulation of neighboring whiskers.

The implications for synthesis of receptive fields in layer 4 are less clear. Still, our findings strongly support the notion that the multi-whisker receptive fields of layer 2/3 cells emerge due to integration at the cortical level, either by integration of independent ascending inputs from layer 4 cells or integration of inputs from layer 2/3 cells of neighboring barrels. Supposing that multi-whisker receptive fields of layer 2/3 cells are inherited from layer 4 cells, we strongly suggest that at the stimulation rate we used in our study (i.e., 10 Hz), no additional adapting mechanisms act in the layer 4 to layer 2/3 pathway. Whether or not adaptation of cortical cells to other types of mechanical stimulation (Lottem and Azouz, 2008; Maravall et al., 2013; Sachidhanandam et al., 2013) exhibits a similar degree of specificity is not clear and remains to be studied.

Nevertheless, our results do not support early convergence of pathways at the thalamic level (Jubran et al., 2016). In such a case, we would expect that a significant portion of whiskers would exhibit strong interactions. The finding that there are asymmetrical interactions, which the depression of the synaptic input cannot explain, raises the possibility that inhibition might participate in the interactions. Still, the validity of our results in awake animals needs to be further studied.

### Possible Mechanisms for Interactions

At least three central mechanisms can be responsible for the interactions between inputs arriving from different whiskers; intrinsic adaptation (Carandini and Ferster, 1997), short-term synaptic depression (Gabernet et al., 2005; Kheradpezhouh et al., 2017), and cross-whisker inhibition (Brumberg et al., 1996; Moore and Nelson, 1998).

Suppose synaptic depression would explain the adaptation of cortical cells to whisker stimulation. In that case, it is reasonable to expect a reduction in response to both whiskers during interleaved stimulation as both inputs from the whiskers ascend through the same pathway. This indeed was the case in ∼20% of interacting cells (6/28 cells). On the other hand, unidirectional interactions in which only one whisker suppresses the other can be attributed to suppression between the pathways. Layer 4 cells efficiently recruit interneurons in layer 2/3 (Armstrong-James et al., 1992; Feldmeyer et al., 2002; Shepherd and Svoboda, 2005; Helmstaedter et al., 2008). Thus, during unidirectional interactions, the principal whisker’s response can activate layer 2/3 interneurons that later shunt the adjacent whisker‘s response. However, to obtain a conclusive decision about the involvement of inhibition in unidirectional interaction, two properties for such inhibition are required. First, the typical time course of IPSCs in layer 2/3 terminates approximately at the same time as the interleaved stimulation ISI (100 ms) (Ling and Benardo, 1995; Heiss et al., 2008) allowing the shunting of following responses. Second, stimulation of the adjacent whisker is not supposed to recruit a significant inhibition since it evokes a relatively weak and slow response. This is feasible if the response of the adjacent whisker arrives from the adjacent barrel, or from the same subset of synapses but is weaker and slower and recruits fewer interneurons (Kapfer et al., 2007). Our data do not meet this condition since the amplitudes or latencies to responses of the affecting whiskers in unidirectional cells are not stronger than those of the affected whiskers (Figure 5B). Another pathway for inhibition can be mediated via the horizontal connections of the neurogliaform cells. During whisker movements, Martinotti cells reduce their tonic inhibition (Gentet et al., 2012), resulting in increased inhibition of neurogliaform cells via horizontal connections (Tamás et al., 2003; Gentet, 2012) that can mediate slow inter-barrel inhibition.

Yet, the inability to predict the interaction level between inputs from different whiskers arriving at the same cell by their amplitude and latency suggests no canonical circuit in layer 2/3 in which the interactions between inputs from two whiskers can be deduced by their response properties. It is possible that future experiments in which recordings will be targeted to specific types of cortical cells using various CRE-lines will reveal more structure across the population in the adaptation pattern and its specificity to whisker identity.

### Possible Implications for Synthesis of Multi-Whisker Receptive Fields in Layer 2/3

During interleaved stimulation, responses were significantly reduced only in 25% (14/56) of inputs evoked by stimulation of the tested whiskers. The reduction in the response of layer 2/3 neurons suggests that some afferents are common to both inputs. This is in favor of the ‘inherited receptive field hypothesis’ which claims that barrel cortex neurons inherit their RF from their thalamic afferents (Goldreich et al., 1999; Lübke et al., 2000; Kwegyir-Afful et al., 2005), in this case from layer 4 cells. On the other hand, in 75% of the inputs, the responses did not show any interactions, which is evidence in favor of the ‘cortical integration hypothesis’ which claims that inputs from adjacent whiskers are transferred via cortico-cortical interactions (Armstrong-James et al., 1991; Fox et al., 2003).

There are (at least) two parallel cortical pathways for the ascending inputs from the adjacent whiskers. One pathway enters layer 2/3 from the adjacent barrel whereas the second arrives from multi-whisker layer 4 barrel cells and thus shares the same synapse with the principal whisker. If the interactions result from depletion of synaptic resources (i.e., synaptic depression Lampl and Katz, 2017), then interactions between inputs evoked by stimulation of two whiskers should depend on the response amplitudes. For example, a very weak response when stimulating one whisker will not affect the response evoked by stimulating the second whisker since it almost does not use mutual resources. On the other hand, input from whiskers with similar amplitudes and latencies probably arriving from the same pathway are expected to show strong interactions. Therefore, it was suggested that the amplitude ratio of responses when stimulating two whiskers and the latency differences can predict the significance of the interactions. On a single cell level, we found that these two predictors were notrelated to the measured interactions (Figure 4). One possible reason for the indistinguishable results might be the small sampled population and lack of cellular identity (barrel- or septa-related).

Another option is the lack of consistency in the exact recording position relative to the center of each barrel column. Few studies point toward an internal barrel map in which the adjacent-whisker would have a broader representation closer to its border (Andermann and Moore, 2006), affecting the response properties and circuitry. Hence the location of the cell within the barrel might set the degree of the interactions. Though we did not find a relation between the amplitude and the interaction, it is possible that a careful mapping of the recording position within the barrel might produce a different result.

Although the source of the interactions could not be revealed in this study, the various types of interactions favor both hypotheses for receptive field integration in layer 2/3.

Many cells did not show any interactions between inputs arriving from the two whiskers, suggesting that layer 2/3 neurons integrate them. Another option for the weak interactions due to synaptic depression during adaptation is the possibility that the firing rate of individual input cells is low, leading to weak synaptic depression (Lampl and Katz, 2017). In that case, input arriving at layer 2/3 is inherited from many layer 4 cells having low firing probability, and the adaptation seen at a layer 2/3 cell results from synaptic adaptation of the previous thalamocortical synapse (Gil et al., 1999; Chung et al., 2002; Jubran et al., 2016) of the stimulated whisker. Yet, because layer 4 cells exhibit multi-whisker receptive fields, we strongly suggest that layer 2/3 cells integrate inputs from different whiskers due to cortio-cortical interactions.

## Conclusion

Our results show complex integration in layer 2/3 cells. For most cells the response to interleaved stimulation of two whiskers mostly shows no interactions, but in a small number of cases, it can result in strong reciprocal interactions or asymmetrical unidirectional interactions. Our results thus combine contrasting studies by supporting both cortico-cortical and inherited receptive fields.

## Data Availability Statement

Custom code in MATLAB and data are available at the Open Science Framework - https://osf.io/f4mvb/.

## Ethics Statement

The animal study was reviewed and approved by the Weizmann Institute Animal Care and Use Committee. Written informed consent was obtained from the owners for the participation of their animals in this study.

## Author Contributions

IL and YK designed the experiments, carried out the analysis, and wrote the manuscript. YK carried out the electrophysiology experiments. Both authors contributed to the article and approved the submitted version.

## Conflict of Interest

The authors declare that the research was conducted in the absence of any commercial or financial relationships that could be construed as a potential conflict of interest.
